# Mitochondrial Network Dynamics in Aging: Cellular Mechanisms, Intercellular Communication, and Their Impact on Tissue Adaptability

**DOI:** 10.3390/ijms27083557

**Published:** 2026-04-16

**Authors:** Luminita Labusca, Teodor Stefan Gheorghevici, Bogdan Puha

**Affiliations:** 1 National Institute of Research and Development in Technical Physics, 700050 Iasi, Romania; 2 Orthopedics and Traumatology Clinic, County Emergency Hospital Saint Spiridon, 700111 Iasi, Romania; steoevici@gmail.com; 3 2 Surgery Department, Grigore T. Popa University of Medicine and Pharmacy, 700115 Iasi, Romania

**Keywords:** mitochondria, aging, mitophagy, intercellular mitochondrial communication, myocardial aging, neurodegeneration, skeletal muscle aging

## Abstract

Beyond their classical role as “cellular powerhouses”, mitochondria are increasingly recognized as dynamic and interconnected networks whose architecture, quality control, and intercellular communication influence cellular and organismal homeostasis. Mitochondrial dynamics—including fusion–fission balance, mitophagy–biogenesis coupling, intracellular organization, and intercellular transfer via tunneling nanotubes, extracellular vesicles, or transient cell fusion—contribute to tissue adaptation and functional decline during aging. Focusing on cardiac muscle, skeletal muscle, and the nervous system, this narrative review synthesizes current evidence describing how aging disrupts mitochondrial network integrity through altered dynamics, impaired organelle positioning and transport, reduced mitophagy, mtDNA instability, and compromised metabolic coupling between cells. These alterations propagate across tissues, limiting energetic flexibility, stress resilience, and regenerative capacity. Building on these mechanisms, we discuss a systems-level perspective in which aging is associated with progressive loss of mitochondrial network coherence rather than solely cumulative molecular damage. Within this framework, mitochondrial connectivity functions as an integrative descriptor of cellular resilience: well-organized networks counteract metabolic perturbations, whereas functionally decoupled networks amplify stress and promote maladaptive aging trajectories. Emerging evidence indicates that physiological and pharmacological interventions, including endurance exercise, caloric restriction or mimetics, fusion-supporting pathways, and mitophagy-enhancing strategies, can partially restore network organization even later in life. Molecular, cellular, and tissue-level insights are integrated to highlight mitochondrial network dynamics as both a mechanistic contributor to aging and a potentially modifiable target for future preventive and therapeutic interventions.

## 1. Introduction

In recent years, mitochondria have increasingly been recognized as complex regulators of cellular function, far beyond their classical role as “powerhouses” of the cell. Their role in cellular metabolism and their implications for cell fate decisions, tissue turnover, immune balance, and consequently for organismal health and disease are increasingly recognized [1]. Our ability to deepen mechanistic insights within subcellular compartments using high-throughput and high-resolution technologies is continuously increasing, in parallel to the capacity to process and integrate large-scale datasets [2]. Such a complex perspective, both analytical and integrative, gradually reveals the hierarchical organization and multilevel interdependence of mitochondrial activity within the cell. Mitochondrial function and interactions can now be resolved at an unprecedented scale to uncover stages of development, cell, tissue, and organ maintenance, as well as contributions to diseased states and organismal decline.

From a systems biology perspective, integrated transcriptomic, proteomic, metabolomic, spatial imaging, and bioinformatic approaches provide a multidimensional view of mitochondrial function [3,4]. They reveal regulatory networks linking mitochondrial dynamics, redox balance, and metabolite exchange with nuclear gene expression and inter-organelle communication. Within this framework, mitochondria act as active nodes coordinating energy flux, stress responses, and signal propagation across cells and tissues. This helps explain how mitochondrial behavior supports homeostasis and adaptation and how its dysregulation contributes to aging and disease. Extending this perspective beyond the single-cell level opens the possibility of modeling how mitochondrial coordination occurs across cells and tissues. Using deep language and computational models, the dynamic mitochondrial networks that orchestrate metabolic states and intercellular communication—potentially shaping emergent physiological or pathological states—could be predicted and further validated.

Current knowledge on mitochondrial function and its alterations during aging is briefly reviewed with emphasis on cardiac muscle, skeletal muscle, and the nervous system, where mitochondrial performance is essential for physiological integrity and age-related decline. The manuscript is structured around mitochondrial connectivity across intracellular, intercellular, and tissue levels as a framework for understanding how mitochondrial dysfunction may contribute to systemic aging.

## 2. Principles of Mitochondrial Network Organization

Mitochondria in living cells function as connected, adaptive networks rather than isolated organelles. Their organization—how mitochondria fuse, divide, move, and renew—shapes energy delivery, redox balance, and the adaptative response. When either network function or its elements become unstable, cells may retain morphological continuity yet lose effective coordination, contributing to aging- and disease-related decline.

### 2.1. Intracellular Mitochondrial Networks

At the intracellular level, mitochondrial networks undergo continuous remodeling through the coordinated balance of fusion, fission, biogenesis, and autophagy/mitophagy, which together determine their organization, turnover, and functional adaptation to cellular demands.

#### 2.1.1. Fusion and Fission Mechanisms

Mitochondrial fusion is mediated by membrane-bound GTPases, with mitofusins (MFN1 and MFN2) regulating outer membrane fusion and optic atrophy 1 (OPA1) controlling inner membrane fusion [5]. Fusion promotes the mixing of mitochondrial content mainly to compensate for deficient units and to increase functional efficiency [6,7]. Fusion is typically adopted during high cellular energetic demand or mild stress, when cells require coordinated distribution of metabolites, mitochondrial DNA (mtDNA), and membrane potential to sustain optimal oxidative phosphorylation (OXPHOS) processes. This state is characteristic of the proliferating, differentiated, or metabolically active cells 2that maintain bioenergetic stability. Reduced protein synthesis or induction of autophagy, particularly via inhibition of the mammalian target of rapamycin (mTOR) or nutrient deprivation, promotes mitochondrial fusion and coordinates mitochondrial activity to support cell viability [8,9]. Beyond protein expression levels, mitochondrial fusion is an energetically costly process. Both outer membrane fusion (mediated by MFN1 and MFN2) and inner membrane fusion (mediated by OPA1) require GTP hydrolysis, linking network maintenance directly to cellular energetic state [10,11]. During aging, declining ATP production and impaired nucleotide regeneration reduce the availability of GTP, thereby limiting the cell’s capacity to sustain fusion even when fusion machinery remains present. Under such conditions, fission becomes energetically favored not only as a quality-control mechanism but also as a passive consequence of energetic insufficiency, during which sustained network fusion becomes metabolically unaffordable.

Fission is governed primarily by dynamin-related protein 1 (DRP1) and its adaptors (Mitochondrial fission protein 1-Fis1, Mitochondrial fission factor-Mff, Mitochondrial Dynamics proteins of 49 and 51 kDa-MiD49/51). Fission allows segregation of dysfunctional segments and facilitates organelle redistribution during mitosis or stress [7]. By fragmenting the network, fission enables the isolation of damaged or dysfunctional mitochondria to further undergo PTEN-induced putative kinase 1 (PINK1/Parkin-mediated mitophagy [12]. The PINK1/Parkin pathway selectively labels mitochondria that have lost membrane potential for autophagic removal. When ΔΨm collapses, PINK1 accumulates on the outer membrane and activates Parkin, which ubiquitylates outer-membrane proteins to recruit autophagy adaptors and initiate engulfment. This pathway prevents damaged, reactive oxygen species (ROS)-producing mitochondria from remaining as “faulty nodes” within the network, thereby protecting energetic coupling and limiting inflammatory signaling. During cell division and differentiation, fission enables equal mitochondrial partitioning between the two daughter cells. Fission is employed as well in response to nutrient deprivation, oxidative stress, or calcium overload [13,14] and mediates apoptotic cell clearance by macrophages [15]. A shift toward excessive fission is observed in apoptotic, senescent, or metabolically altered states, contributing to mitochondrial depolarization and impaired bioenergetic efficiency [16].

The interplay between fusion and fission ensures metabolic flexibility and quality control, linking mitochondrial morphology to cellular energetic and redox states. With aging and disease, disruption of this balance undermines network integration and can lead to functional decoupling of the mitochondrial reticulum.

#### 2.1.2. Functional Fragmentation in Aging

Mitochondrial network deterioration during aging is more than a morphological disruption. While increased fission and fragmentation are frequently observed, aged tissues also display hyper-fused, swollen, or toroidal (“donut-shaped”) mitochondria, as well as networks that remain morphologically continuous yet functionally compromised. These configurations likely represent stress-adaptive or pre-degenerative states rather than preserved network health. Loss of mitochondrial connectivity during aging is often functional rather than purely topological, reflecting impaired energy conduction, quality-control responsiveness, and signal integration despite apparent structural continuity.

Mitochondrial connectivity must be considered at two distinct architectural levels. Outer mitochondrial membrane fusion governs reticular continuity and physical networking, whereas inner membrane fusion and cristae junction integrity determine electrical conduction, proton-motive force distribution, and respiratory efficiency [17]. Emerging evidence indicates that in aged tissues, mitochondria may remain connected at the outer membrane level while exhibiting profound inner membrane disorganization, including cristae shortening, vesiculation, or junction collapse. Such alterations uncouple mitochondrial inner membrane potential (ΔΨm) propagation and ATP distribution, even if morphological continuity may be preserved [18]. Functional decoupling is not only a matter of impaired conduction within an otherwise continuous reticulum but is also a progressive shift in both the number and functional quality of mitochondrial nodes.

#### 2.1.3. Mitochondrial Turnover, Quality Control and Energy Sensing

Both the number and the functional quality of mitochondria—which can be viewed as nodes within a network—are key determinants of collective mitochondrial behavior. Node density and competence are maintained by the coupled processes of mitophagy (selective removal) and mitochondrial biogenesis (renewal), together preserving coherence within the dynamic reticulum. Damaged or depolarized mitochondria are removed through the PINK1–Parkin pathway, whereas renewal depends on PGC-1α–driven biogenesis. The balance between these processes determines the pool of functional nodes, network resilience, and bioenergetic stability [19,20].

Energetic and nutrient states influence mitochondrial turnover through the AMPK–mTORC1 axis. Under energy deficit, AMPK activates ULK1 to promote autophagy/mitophagy and supports biogenesis via the SIRT1–PGC-1α pathway [21,22]. In contrast, mTORC1 suppresses ULK1-dependent autophagy under nutrient sufficiency and adjusts mitochondrial gene expression in anabolic states [23]. At the transcriptional level, PGC-1α co-activates NRF1/NRF2 and induces TFAM, supporting mtDNA replication/transcription and replenishment of the mitochondrial pool [24,25]. Receptor-mediated mitophagy (e.g., FUNDC1) is transcriptionally coupled to PGC-1α/NRF1 signaling, linking degradation and renewal within a quality-control loop [26]. PINK1/Parkin-mediated mitophagy, PGC-1α biogenesis, and AMPK/mTOR-regulated signaling operate as an integrated homeostatic system aligning the mitochondria network with the cell state [27,28]. Mitophagy is activated during energetic stress, hypoxia, oxidative injury, mtDNA damage, or cell-cycle arrest, conditions in which ROS-producing organelles accumulate. PINK1/Parkin and receptor-mediated pathways (BNIP3, NIX, FUNDC1) remove dysfunctional mitochondria, therefore limiting ROS [29,30].

**Figure 1:**
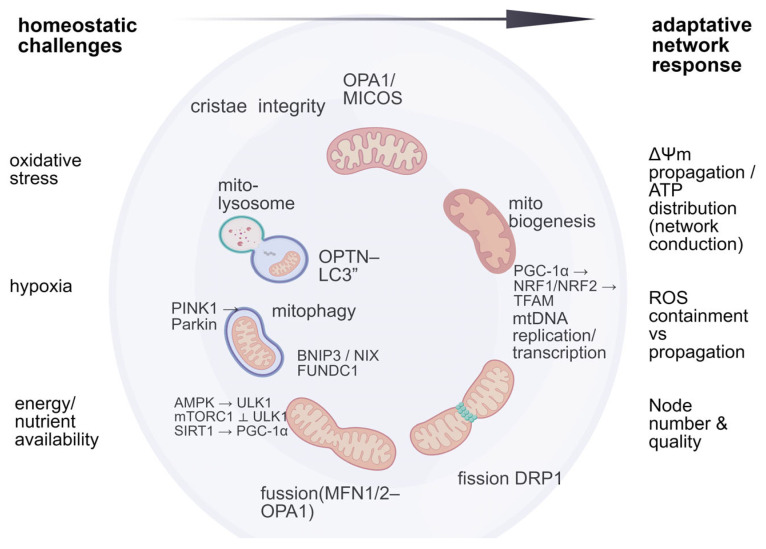
Intracellular mitochondrial network homeostasis integrates dynamics and turnover. Fusion (MFN1/2–OPA1) and fission (DRP1) remodel the reticulum, while quality control removes damaged nodes via PINK1/Parkin- and receptor-mediated (BNIP3/NIX) mitophagy; energy/nutrient sensing (AMPK→ULK1; mTORC1 ⟂ ULK1) coordinates turnover with mitochondrial renewal (PGC-1α→TFAM biogenesis) ΔΨm propagation, ATP distribution, ROS containment, node number/quality maintenance. Arrow designates activation (composed by authors using BioGDP.com).

In stem or progenitor cells, mitophagy supports quiescence or early differentiation by maintaining a glycolytic, low-ROS profile [31,32]. Conversely, biogenesis predominates during stress recovery, proliferation, or differentiation, when increased OXPHOS capacity is required. PGC-1α–NRF1/2–TFAM signaling is induced downstream of AMPK/SIRT1 to restore mitochondrial content after mitophagic clearance and to match increased energetic demand [33,34,35]. Mitophagy and biogenesis form a physiological continuum in which stress promotes selective removal, whereas recovery and anabolism trigger PGC-1α–mediated renewal, restoring network quality and density [36]. Disruption of this balance is a hallmark of aging and contributes to mtDNA instability, cristae alterations, and impaired energy metabolism. Accordingly, healthy cellular homeostasis depends on an intracellular mitochondrial network capable of adaptive remodeling through fusion, fission, biogenesis, and autophagy/mitophagy (Figure 1).

### 2.2. Intercellular Mitochondrial Communication

Mitochondria can cross cellular boundaries and interact with surrounding cells and extracellular matrix components. mtDNA or even entire mitochondria can be transported via tunneling nanotubes (TNTs), microvesicle- or exosome-mediated transfer, as well as during cell fusion events.

#### 2.2.1. Tunneling Nanotubes (TNTs)

Tunneling nanotubes (TNTs) are actin-rich membranous protrusions connecting donor and recipient cells, along which entire mitochondria or mitochondrial fragments can be mobilized [37,38]. TNTs vary in diameter (from ~50 nm up to ~1 µm) and mediate either direct cytoplasmic continuity or close apposition between donor and recipient cells [39].

TNT-based mitochondrial transfer mediates the antimicrobial effect of human MSC administration in an in vivo model of acute bacterial pneumonia, improving alveolar macrophage bioenergetics and enhancing their phagocytic ability [40,41]. TNT-transferred mitochondria influence multiple physiological and pathological processes, including immune and reparative responses, neural plasticity, and malignant progression with acquired resistance to therapy [42,43]. TNT transfer is typically initiated by donor cells in response to mitochondrial dysfunction or oxidative stress in recipient cells. Donor cells activate RalA–M-Sec–exocyst signaling together with actin polymerization to form TNTs. Mitochondria are subsequently recruited through the MIRO1–TRAK–myosin/kinesin transport machinery and transported along actin- or microtubule-based tracks within the TNT toward the recipient cell. There, integration with the endogenous mitochondrial network can restore membrane potential, oxidative phosphorylation, and ATP production [44], being increasingly discussed as a potential therapeutic target [45].

To date, there is no evidence of TNT mitochondrial transfer to rescue senescent cells in vivo; rather, senescent phenotype appears to employ this mechanism to propagate to surrounding cells. Life imaging tracking was able to document that senescent cells act rather as mitochondrial donors in a mammalian target of rapamycin (mTOR) and of small GTP-ase CDC42-dependent manner, contributing to spreading senescence-associated phenotype (SASP) to surrounding cells and, potentially, tissues [46].

#### 2.2.2. Extracellular Vesicle-Mediated Mitochondrial Transfer

Mitochondria can circulate as well via EVs containing the entire organelle or components (mtDNA or proteins). Mitochondria-containing EVs can be released under physiological stress and subsequently internalized by recipient cells through endocytosis. After uptake, mitochondrial cargo contributes to restoring or reshaping recipient-cell bioenergetics, for example, by supplementing respiratory capacity or supporting recovery from mitochondrial dysfunction. In support of this concept, mitochondria-containing EVs derived from autologous stem cell–derived cardiomyocytes were reported to improve ischemic myocardial energetics, at least in part through functional integration and fusion with the endogenous mitochondrial network of recipient cells [47]. Conversely, stressed MSCs can also export mitochondria via EVs; under oxidative stress conditions, MSCs have been shown to package and release partially depolarized mitochondria in EVs, pointing towards a context-dependent role for EV-mediated mitochondrial trafficking in intercellular adaptation and mitochondrial quality control.

EV-mediated mitochondrial transfer involving MSCs is increasingly recognized as bidirectional. MSCs can donate functional mitochondria to metabolically compromised cells, restoring OXPHOS and supporting tissue repair [48,49], but stressed or damaged niche cells may also transfer dysfunctional mitochondria to MSCs. Such reverse transfer induces mitochondrial overload, metabolic stress, and premature senescence in recipient stem cells, potentially limiting long-term regenerative capacity [50,51]. Mitochondrial exchange between stem cells and their niche therefore appears to be a double-edged process, supporting repair or propagating aging signals depending on organelle quality.

EV-based export may also serve as a mechanism for clearance of dysfunctional mitochondria. Under oxidative stress, MSCs export depolarized mitochondria within EVs, which are subsequently recycled by macrophages, restoring intracellular bioenergetics [52,53]. Declining plasma levels of mtDNA-containing EVs correlate with advancing age and have been proposed as a biomarker of health status in middle-aged and older individuals [54]. However, standardized methods for sampling, isolation, characterization, assessment of mitochondrial origin, and biomarker validation are still lacking [55].

#### 2.2.3. Mitochondrial Exchange During Cell–Cell Fusion and Other Exchange Mechanisms

A third mechanism of intercellular exchange is cell–cell or heterotypic fusion, during which cytoplasmic organelles, including mitochondria and mitochondrial DNA, are shared [56]. Human multipotent adipose-derived stem cells (hMADS) and human bone marrow-derived stem cells (BMSCs), but not human fibroblasts, were shown to reprogram fully differentiated mouse cardiomyocytes in vitro through cell fusion and mitochondrial transfer [57], suggesting a regenerative mechanism that may be conserved across species. Cell-fusion-mediated mitochondrial transfer from donor BMSCs also improved Müller glial cells in vitro and in a rat model of degenerative retinopathy, reducing oxidative stress and improving function and vision [58]. Cellular fusion events have also been reported to induce senescence through nuclear/genomic stress in viral oncogenesis and tumor metastasis [59,60]. At present, however, no evidence shows that cell fusion-based mitochondrial trafficking either promotes or reverses senescence.

**Figure 2:**
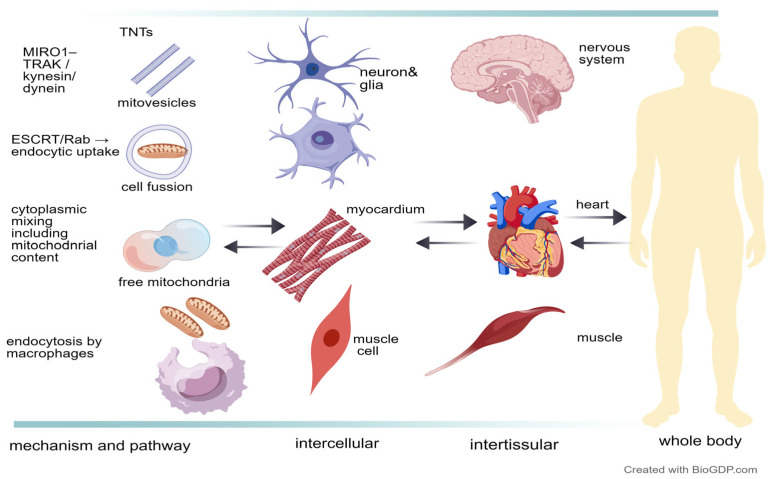
Intercellular mitochondrial exchange occurs mainly via tunneling nanotubes (TNTs), mitochondria-containing extracellular vesicles (“mitovesicles”), cell–cell fusion, and release/uptake of free mitochondria. TNT formation involves actin remodeling pathways (RalA–M-Sec/TNFAIP2–exocyst), while mitochondrial mobilization commonly relies on the MIRO1/2–TRAK1/2 adaptor complex linking mitochondria to motor proteins (kinesin/dynein). EV-associated transfer depends on vesicle biogenesis and trafficking regulators (e.g., ESCRT components, Rab GTPases), and recipient-cell uptake occurs primarily via endocytic routes. Functional outcomes depend on cargo quality and context, ranging from metabolic support to propagation of stress signals; mitochondrial trafficking and signaling may extend beyond intercellular exchange (examples shown include neurons, glia, cardiomyocytes, and skeletal muscle cells) to contribute to inter-tissue, inter-organ, and potentially whole-body coordination. Arrows indicate that exchange can be bidirectional. Figure created using BioGDP.com.

Less common routes of intercellular exchange include direct release of free mitochondria into the extracellular space, followed by uptake by recipient cells through phagocytosis or endocytosis. Intact mitochondria have been detected in conditioned media and subsequently internalized by neighboring cells, mainly through fluid-phase or nonspecific endocytosis; some escaped the endosomal compartment and may have integrated into the host mitochondrial network [61]. Release of dysfunctional mitochondria has also been described in some cell types, such as neurons and adipocytes, as part of the quality control process, followed by macrophage clearance [62]. Intercellular mitochondrial exchange has been described as a mode of communication that can rescue stressed neighboring cells by restoring bioenergetic function and may extend beyond local interactions to inter-tissue and inter-organ coordination, with possible whole-body implications (Figure 2).

## 3. Tissue-Specific Impact of Mitochondrial Networking in Aging

In the following, several tissues characterized by high energy requirements that infer significant and organismal-level decline during aging, will be presented from the perspective of mitochondrial networking. In cardiac tissue, mitochondrial network coherence is essential for continuous ATP supply and excitation–contraction coupling. Skeletal muscle is one of the most energy-demanding body structures and tends to fail with age, exhibiting pronounced mitochondrial plasticity, allowing network remodeling in response to workload and metabolic stress. In the nervous system, mitochondrial network organization supports synaptic transmission, axonal maintenance, and neuron–glia coupling; age-associated fragmentation and impaired trafficking compromise local ATP supply and calcium buffering at synapses, contributing to functional decline and neurodegenerative susceptibility.

### 3.1. Cardiac Muscle

The heart is one of the most mitochondria-rich organs, reflecting its continuous and intense energetic demand. Cardiac mitochondria occupy nearly one-third of the cell volume and are organized into distinct subpopulations—subsarcolemmal and interfibrillar—each specialized for spatially coordinated energy supply. During aging, structural disorganization, oxidative stress, and altered dynamics of fission and fusion compromise mitochondrial efficiency and calcium handling, leading to impaired contractility and increased susceptibility to ischemic heart injury [63].

#### 3.1.1. Mitochondrial Network Fragmentation in Aging Cardiac Muscle

Effective intracellular integration and intercellular coupling of cardiomyocytes are required for proper cardiac function and rhythmic contraction. In adult cardiomyocytes, mitochondria form an electrically coupled reticulum segmented into inter-mitochondrial junction (IMJ)-linked sub-networks that conduct ΔΨm and redistribute ATP supply on a beat-to-beat basis while disconnecting damaged regions as a “circuit-breaker” [64,65]. With aging, this network shifts toward fragmentation due to altered dynamics (reduced MFN2/OPA1-mediated fusion relative to DRP1-driven fission) and cristae destabilization, impairing energy conduction and favoring ROS propagation and loss of contractility [66,67]. OPA1/MICOS (Mitochondrial Contact site and Cristae Organizing System)-dependent inner-membrane and crista junction integrity is particularly important for maintaining networked respiration. Different degrees of perturbations of these systems are linked to cardiac dysfunction in both experimental and clinical settings. OPA1 is involved in the occurrence and progress of dilatative cardiomyopathy, governing fusion–fission imbalance by disrupting mitochondrial inner membrane fusion, as well as the modified cristae structure [68]. Cristae structure and dynamics have been recently identified as a modality to fine-tune oxidative phosphorylation. Cristae vesicles could trap protons as well as ATP, influencing their availability to cytosol, while the fusion of cristae to IBM increases OXPHOS exchanges [69].

With advanced age, cardiomyocytes exhibit downregulation of the MFN2/OPA1 axis as well as relative upregulation/activation of DRP1 involved in fission and impaired cristae architecture. This leads to the build-up of a cardiac muscle that is more prone to impaired excitation–contraction energetics, diastolic/systolic dysfunction, and vulnerability to stress [70]. Excess DRP1-dependent fission promotes apoptotic signaling and pathological remodeling, contributing to heart-failure phenotypes [71]. Mitochondrial function and positive fusion/fission balance can be increased by targeting the sirtuin1 (SIRT1)/AMPK/Drp1 pathway to restore cardiac function [72].

#### 3.1.2. Dysfunctional Mitochondria in Aging Cardiac Muscle

Mitochondrial networks populated with dysfunctional elements contribute to decreased connectivity. In normal adult hearts, the PINK1/Parkin mitophagy cascade is triggered by loss of mitochondrial membrane potential: depolarization blocks PINK1 import and PARL-mediated cleavage, causing PINK1 to accumulate on the outer mitochondrial membrane, where it recruits and activates the E3 ligase Parkin by phosphorylating both Parkin and ubiquitin [73]. The resulting ubiquitin chains are read by autophagy receptors such as Optineurin, which bind ubiquitinated outer mitochondrial membrane (OMM) proteins and simultaneously engage microtubule-associated protein 1A/1B-light chain 3 (LC3) via LC3-interacting region (LIR) motifs, thereby coupling damaged mitochondria to nascent autophagosomes [74]. Aging reduces effective mitophagy signaling, such as the PINK1–Parkin axis, allowing dysfunctional mitochondria to persist within the network [75]. An experimental increase in mitophagy has been demonstrated to improve cardiac function. Urolithin A (UA), a mitophagy activator, enhanced cardiac mitophagy and improved systolic/diastolic function in naturally aged and heart-failure mouse models; electron microscopy and molecular markers indicated restored mitochondrial quality control [76]. In a clinical observational study, a 4-month duration of oral urolithin supplementation in healthy older adults was associated with reduced validated biomarkers of cardiovascular risk (plasma ceramides) [77,78,79,80,81,82].

#### 3.1.3. Impact of Dysfunctional Mitochondrial Networks on Cardiac Function

Mitochondrial autophagy declines in the aging heart. DNA alterations, a hallmark of aging [83], impair mitochondrial network connectivity. Aging hearts accumulate mtDNA lesions and heteroplasmy, which depress respiratory chain function, reduce ΔΨm, and destabilize cristae, thereby compromising reticular coupling across cardiomyocyte mitochondrial subnetworks. The result is regional conduction disturbance, increased intracellular ROS, and reduced contractile reserve. In Polg mutant mice, age-related mtDNA mutations are associated with myocardial hypertrophy, systolic and diastolic dysfunction, and increasing fibrosis, supporting a role for mtDNA damage in cardiomyopathy [84].

Since inter-mitochondrial junctions enable rapid ΔΨm conduction, mtDNA-driven loss of membrane potential or crista integrity can decouple subnetworks and impair myocardial contractility. Nutritional, pharmacological, and genetic interventions that reduce mtDNA heteroplasmy restore mitophagy, dynamics, and biogenesis, delaying age-related cardiovascular degeneration [85].

**Figure 3:**
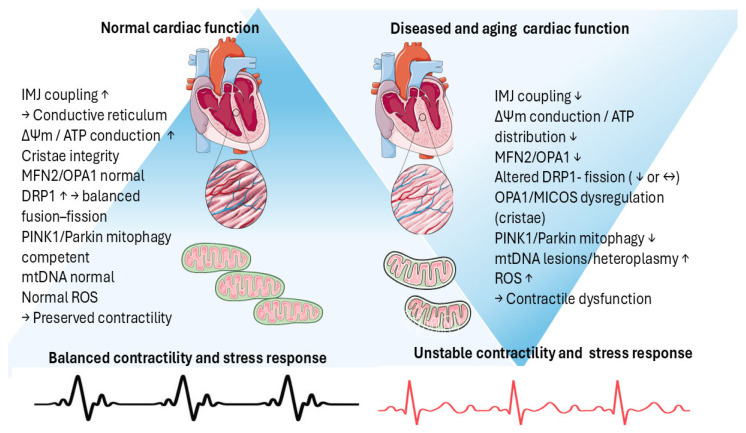
Cardiac mitochondrial network remodeling in aging. Compared with normal adult cardiac function, aging is characterized by reduced IMJ-mediated coupling and ΔΨm conduction, a shift toward DRP1-biased fission reduced MFN2/OPA1 support, cristae destabilization (OPA1/MICOS), impaired PINK1/Parkin-dependent mitophagy, mtDNA lesion/heteroplasmy accumulation, and increased ROS, reducing contractile reserve and stress tolerance. Figure created by authors with Elsevier free art (SMART).

Adult human cardiomyocyte renewal is very slow—about 1% per year at age 25, falling to about 0.45% per year by age 75—so damaged cells persist for decades, and defects in mitochondrial networking and quality control accumulate rather than being diluted by cell replacement. This favors functional decoupling of the mitochondrial network through altered dynamics (MFN2/OPA1, ↑DRP1), mtDNA lesion and heteroplasmy build-up, and declining mitophagy, allowing dysfunctional organelles to persist [86]. Together, these disturbances reduce ATP reserve, increase intracellular ROS, and impair beat-to-beat contractile performance, predisposing the aging heart to systolic/diastolic dysfunction and stress intolerance (Figure 3).

### 3.2. Skeletal Muscle

Skeletal muscle represents another paradigm of mitochondrial plasticity, as its metabolic phenotype continuously adapts to mechanical load, nutrient availability, and hormonal and metabolic cues. This dynamic connectivity depends on a delicate balance between fusion and fission, as well on the quality of mitochondrial elements within the network. Similar to cardiac muscle, synchronized mitochondrial membrane depolarization in skeletal muscle depends on a continuous network; thus, even minor fragmentation can lead to significant energetic and contractile deficits.

#### 3.2.1. Mitochondrial Network Disruption in Skeletal Muscle

With advancing age, the decline in mitochondrial biogenesis, mitophagy efficiency, and respiratory capacity contributes to age-related sarcopenia and decreased endurance. Mitochondrial DNA mutations and the accumulation of dysfunctional organelles disrupt energy homeostasis and redox signaling, amplifying anabolic resistance and inflammation and perturbing mitochondrial intracellular and intercellular networking.

In the young adult skeletal muscle, mitochondria form a reticular network extending along myofibrils and across sarcomeres, ensuring efficient distribution of ATP, metabolites, and calcium [87]. This interconnected network, known as the mitochondrial reticulum, enables rapid energy distribution by providing a conductive pathway for the proton-motive force. Coordinated mitochondrial depolarization and conduction via the mitochondrial reticulum have been proposed as the main modality of energy distribution within skeletal muscle [88]. The proximity of the mitochondrial reticulum to calcium stores in the sarcoplasmic reticulum enables bidirectional signaling: calcium stimulates ATP production, while ATP is used during calcium removal. This bidirectional communication ensures that energy supply is closely matched to the muscle’s contraction and relaxation needs, optimizing muscle performance [89].

#### 3.2.2. Decline in Structural and Functional Mitochondrial Networking

With advancing age, both structural continuity and functional coupling within the mitochondrial reticulum deteriorate. Electron microscopy and high-resolution 3D imaging have shown fragmented, swollen, and spatially isolated mitochondria in aged myofibers, in contrast with the highly interconnected networks seen in young muscle [90]. These morphological changes parallel functional decline: aged mitochondria produce ATP less efficiently and are more susceptible to oxidative stress, contributing to muscle atrophy and sarcopenia [91]. Mitochondrial organization depends not only on morphology but also on interconnectivity. Reduced inter-mitochondrial contacts and altered cytoskeletal anchoring limit calcium and metabolite diffusion, further decoupling bioenergetic domains. Studies in human and animal muscle further show that aging disrupts the microarchitecture linking excitation–contraction (EC) coupling to mitochondrial function. Muscle biopsies from sedentary older adults show a 40–50% reduction in calcium-release units (CRUs) compared with young individuals, a loss associated with impaired specific force generation [92]. Sarcopenic mice and human muscle biopsies show parallel declines in total mitochondrial number and in CRU–mitochondrial pairs, indicating that mitochondria progressively lose their strategic positioning near SR Ca^2+^ release sites with age [93,94]. This uncoupling is accompanied by reduced SR Ca^2+^ release, impaired mitochondrial Ca^2+^ uptake, and increased oxidative stress, underscoring the importance of CRU–mitochondrial alignment for coupling Ca^2+^ signaling to ATP synthesis and buffering ROS/RNS [95,96]. Disorganization of the mitochondrial network lowers the respiratory control ratio, slows ATP turnover, and delays recovery, all features of sarcopenic decline [97,98]

ATP turnover declines with age, correlating with slower phosphocreatine (PCr) recovery kinetics (kPCr) after exercise, lower oxidative capacity reduced cardiorespiratory fitness, and reduced donor muscle strength [99,100].

**Figure 4:**
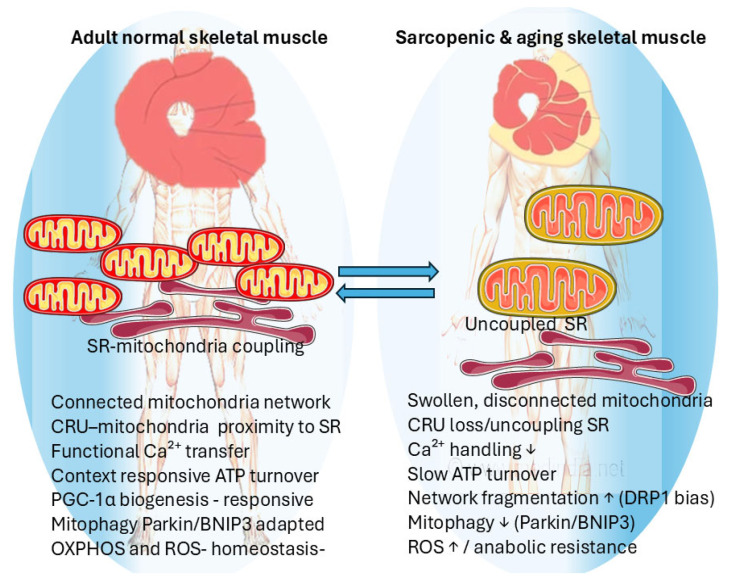
Skeletal muscle mitochondrial network remodeling in aging. Left: In adult healthy skeletal muscle, mitochondria form a connected network positioned in close apposition to the sarcoplasmic reticulum (SR)/calcium release units (CRUs), supporting efficient Ca^2+^ transfer, context-responsive ATP turnover, and preservation of OXPHOS and ROS homeostasis. Mitochondrial content and quality are maintained by the PGC-1α biogenesis axis and mitophagy (Parkin/BNIP3). Right: In sarcopenic and aging muscle, mitochondria become swollen and disconnected, with CRU/SR loss or uncoupling, reduced Ca^2+^ handling, slowed ATP turnover, network fragmentation (DRP1-biased remodeling), reduced mitophagy (Parkin/BNIP3), and increased ROS, contributing to anabolic resistance and decline.Ascendent arrow designates increase, descendent arrow designates decrease. Figure created by authors with Elsevier free art (SMART).

Aging skeletal muscle decreases intracellular connectivity between the mitochondria nodes of the network. Mitochondrial fusion mediators MFN2 and OPA1were found to be downregulated in aging muscle, with relative upregulation or hyperactivation of DRP1, contributing to excessive fission, inducing network fragmentation and loss of cristae integrity [101]. In adult mice, specific deletion of Drp-1 induces ER stress, which has as a consequence catabolic muscle loss and systemic senescence mediated by the unfolded protein response (UPR) and FoxOs signaling. In mice with double deletion of Opa1 and Fgf21 genes, the aging phenotype was completely reversed. Sedentary but not physically active subjects display age-correlated decline of OPA-1 in skeletal muscle biopsy. [102]. The reduced capacity for complementation between partially damaged mitochondria amplifies local oxidative stress and mtDNA instability. In this “negative feedback loop,” mitochondria are a major source of ROS, further impairing mtDNA [103]. Not only interconnectivity but also mitochondrial number and quality decline in aging muscle. Transcriptomic analyses reveal reduced expression of PGC-1α, TFAM, and NRF1/2, the main regulators of mitochondrial biogenesis, thereby limiting renewal of network components [104]. Reduced biogenesis is associated with impaired skeletal muscle performance, decreased respiratory capacity, and accumulation of dysfunctional mitochondrial elements. However, targeted overexpression of biogenesis regulators does not appear to have a straightforward restorative effect. In aging mice, skeletal muscle-specific overexpression of PGC-1α enhanced mitochondrial biogenesis and supported anabolic signaling but also induced oxidative stress and inflammatory responses. In addition to fragmentation and swelling, aging muscle fibers may show focal mitochondrial clustering, reflecting disruption of the normally ordered interfibrillar distribution of mitochondria. Such aggregates likely result from cumulative alterations in fusion–fission balance, impaired mitochondrial transport, and declining quality-control mechanisms, ultimately contributing to local heterogeneity in membrane potential and reduced bioenergetic coupling within the mitochondrial network [105] (Figure 4).

#### 3.2.3. Altered Mitochondrial Stress Response in Aging Muscle

NF-κB is a stress- and cytokine-responsive transcription factor that coordinates inflammatory and pro-survival gene programs. In the canonical pathway, NF-κB dimers are retained in the cytosol by IκB proteins; inflammatory cues activate the IKK complex, leading to IκB phosphorylation and degradation, NF-κB nuclear translocation, and induction of targets such as TNF-α and other inflammatory mediators. In aging skeletal muscle, persistent NF-κB activation is commonly associated with oxidative stress and mitochondrial damage signaling and correlates with impaired mitochondrial quality control and anabolic resistance.

Consistent with this profile, aged muscle shows increased protein carbonylation and altered expression of stress-response and repair markers, including increased IκB-α, NF-κB, TNF-α, SOD2, and NRF2, together with reduced expression of the DNA repair enzyme OGG1. Notably, a comparable inflammatory/oxidative signature was reported in the hippocampus despite PGC-1α overexpression being restricted to skeletal muscle, supporting a context-dependent and potentially systemic impact of muscle mitochondrial remodeling during aging [106]. In line with this, muscle PGC-1α overexpression enhances the biogenic response, whereas its absence attenuates exercise-induced mitochondrial remodeling [107,108,109,110,111].

Defects in mitochondrial quality control further aggravate this inflammatory state. Reduced mitophagy can lead to accumulation of fragmented organelles due to impaired PINK1–Parkin signaling and defective lysosomal flux, contributing to decreased myofibrillar area and muscle atrophy [112,113,114,115,116]. Persisting dysfunctional mitochondria release ROS and mitochondrial DAMPs, reinforcing NF-κB–driven inflammatory cascades and further promoting anabolic resistance in aging skeletal muscle [117,118,119]. Taken together, aging muscle progressively loses mitochondrial network connectivity—both morphologically and functionally. Energetic inefficiency, redox imbalance and chronic low-grade inflammation lead to loss of function and motor impairment.

### 3.3. Nervous System

Neurons are among the most energy-dependent cells in the body, yet they possess minimal regenerative capabilities and rely on a finely tuned mitochondrial infrastructure to sustain their lifelong function. Synaptic transmission, long-distance axonal transport, calcium buffering, and adaptive stress responses all depend on the structure, organization, mobility, and connectivity of the mitochondrial network.

#### 3.3.1. Mitochondrial Network Disruption in the Aging Nervous System

With advancing age, the tightly regulated processes that maintain mitochondrial networking in the nervous system gradually deteriorate. Mitochondria become less efficient, more fragmented, and increasingly unable to meet the bioenergetic demands of neuronal circuits. As a result, neurons accumulate oxidative damage, display impaired proteostasis, and lose synaptic integrity [120,121]. Although these alterations are well described in neurodegenerative disorders, they are not exclusive to them but represent broader age-related vulnerabilities affecting neuronal resilience and function.

Aging neurons shift from elongated, interconnected mitochondrial networks toward shorter, rounded, and fragmented organelles. Longitudinal imaging in *C. elegans* showed that neuronal aging is accompanied by progressive mitochondrial fragmentation associated with functional decline, whereas maintenance of fused networks delays behavioral deterioration [122]. Similar changes are seen in mammals: ultrastructural studies in aged mouse hippocampus revealed shortened, spherical mitochondria with disrupted cristae compared with the elongated profiles found in young animals [123,124,125]. These changes reflect an altered fission–fusion balance rather than morphology alone. In the aged hippocampus, increased Drp1-Ser616 phosphorylation together with reduced Mfn2 expression is associated with higher fragmentation indices, impaired mitochondrial respiration, and memory decline. Senescent cortical astrocytes and astrocytes from aged mouse brain likewise show increased numbers of small, fragmented mitochondria, reduced membrane potential, and marked DRP1 upregulation, indicating that aging shifts mitochondrial dynamics toward fragmentation in both neurons and glia, with likely consequences for metabolic support of neural circuits [126].

In postmortem samples from patients with Alzheimer’s disease, electron microscopy revealed swollen and fragmented mitochondria with damaged cristae in both pre- and postsynaptic compartments, together with reduced synaptic vesicle content in neurons and glia across several brain regions [127]. Because direct neural tissue access is limited, studies have also examined more accessible cells. Fibroblasts from patients with Alzheimer’s disease showed reduced average mitochondrial length, a higher proportion of short mitochondria, and altered OPA1/MFN1 processing, consistent with a fragmented network compared with age-matched controls [128]. Increased proportions of morphologically abnormal, ROS-producing mitochondria, together with lysosomal/autophagic defects, were also reported in these cells [129], further supporting the view that fragmentation and accumulation of damaged mitochondria are common features of age-related neurodegenerative disease.

#### 3.3.2. Impaired Mitochondrial Axonal Trafficking

In *Caenorhabditis elegans* mechanosensory neurons, mitochondrial trafficking in distal processes was found to decrease progressively from early adulthood, in parallel with late-life reductions in axonal mitochondrial density and resistance to oxidative stress [130]. In vivo imaging studies (multiphoton imaging) of retinal ganglion cell axons in mice showed that between adulthood (4 months) and old age (23–25 months), the duration and distance of mitochondrial runs and the duty cycle of movement progressively decline, and mitochondria-free gaps along the axon and the proportion of shorter mitochondria increase, indicating less efficient delivery of organelles to distal axonal segments even though the number of transported mitochondria per axon is conserved. The decline is worsened by the co-existence of specific diseases (such as glaucoma) [131]. In sciatic nerve and central nervous (optical nerve, hippocampal region) explants from aging mice, quantitative live imaging demonstrated that the fraction of motile axonal mitochondria and other cargoes declines in two age-dependent phases, one in early adulthood and another one occurring in old subjects, supporting the view that reduced transport is not a continuous, gradual process. Furthermore, at least in the peripheral nervous system, triggers of regeneration were able to increase axonal transport, suggesting that this might be an environment-responsive process and that axonal transport is not obligatorily diminished by age but rather by loss of functional demand. The interference and impact of neurodegenerative diseases on this process remain to be further evaluated [132].

Indirect data from human postmortem samples regarding motor protein levels, localization, and mitochondrial localization also support the fact that in the absence of neurodegenerative disease, adaptive changes in retrograde transport machinery occur rather than clear failure. Dynein adaptor dynactin-P50, βAPP, synaptophysin, and phospho-tau were quantified in hippocampal tissue from cognitively intact individuals of different ages and from AD patients. In neuropathology-free aged cases, advanced age was associated with coordinated increases in dynactin-P50 and βAPP in pyramidal neurons, interpreted as an adaptive up-regulation of retrograde transport capacity during normal aging. In contrast, AD brains showed reduced overall dynactin-P50 and βAPP levels and misplacement to dystrophic neurites and plaques, suggestive of the breakdown of retrograde axonal transport and cargo recycling in disease [133]. Post-mortem frontal cortex from AD patients and age-matched cognitive unaffected controls revealed that kinesin light chain (KLC) and dynein intermediate chain are significantly reduced in AD tissue, with concomitant changes in βAPP and phospho-tau distribution. These can be regarded as evidence for impaired axoplasmic (axonal) transport in human AD cortex rather than an epiphenomenon [134]. In another study, perikarya and neurites from AD patients have depleted or mislocalized mitochondria despite preserved individual mitochondrial volume. These findings are similar to those seen in a tauopathy mouse model and are reversible when soluble tau expression is suppressed, implying the tau-linked disruption of mitochondrial trafficking/axonal transport in AD brain [135]. Synaptic mitochondrial dynamics disruption in AD patients might be cortical-region-dependent, as revealed by EM studies also reporting aberrant mitochondrial morphology, multivesicular bodies within synapses, and diminished synapse apposition length proximal to AD plaques [136].

Currently, direct axonal trafficking studies in animal models, as well as indirect reports from animal and human samples, suggest age-related adaptive changes in axonal transport. Neurodegenerative diseases (particularly reflecting AD-related changes, which appear to be the most well studied) are accompanied by failure of mitochondrial distribution around the synaptic region, and reduced levels and anomalies in phosphorylation of transport proteins are related to structural changes induced by tau plaque accumulation.

#### 3.3.3. Region-Dependent Effects of Mitochondrial Impairment in the Nervous System

Deficient mitophagy, a common mechanism of network impairment during aging, appears to differently impact various CNS regions while acquiring a different profile in neurodegenerative diseases. In animal models, mitophagy reporter mice have provided the clearest evidence that neuronal mitophagy declines in specific brain regions during aging. In mt-Keima reporter mice, lysosome-engulfed mitochondria were detected in several tissues; aging was associated with a marked reduction in basal mitophagy, in hippocampal dentate gyrus neurons of 21-month-old versus 3-month-old subjects, supporting the fact that “normal” aging does not imply overspread defective mitophagy but rather a dynamic adaptive process. Longitudinal mapping with dual autophagy/mitophagy reporters showed that neuronal mitophagy in the mouse brain follows cell- and region-specific trajectories: mitophagy can increase through adulthood and mid-life in hippocampal and cortical circuits and then decline in old age (e.g., CA1 pyramidal neurons, dentate gyrus parvalbumin interneurons), whereas macro autophagy shows an earlier and more global age-related decrease [137].

In aged mice lacking the phospholipase iPLA2β, age-dependent cognitive decline in the prefrontal cortex is accompanied by reduced PINK1/Parkin-dependent mitophagy, decreased LC3-II and increased p62, and accumulation of damaged mitochondria in cortical neurons, partially rescued by pharmacological mitophagy activation [138].

These data support a model in which physiological aging produces a nuanced remodeling of neuronal mitophagy—declining in some neuronal populations and brain regions but not globally extinguished—a possible adaptation of mitochondrial network functioning for preserving more frequently utilized neural groups’ functioning.

#### 3.3.4. Normal Versus Pathological Aging in the Nervous System

In AD, mitophagy defects in neurons have a mechanistically distinct profile. Hippocampal and cortical neurons from AD mouse models and human AD brain tissue exhibit reduced mitophagy flux, with altered levels of PINK1/Parkin-pathway proteins, accumulation of mitochondrial fragments and autophagosomes, and impaired clearance of damaged mitochondria. Pharmacological enhancement of mitophagy (e.g., urolithin A, NAD^+^ boosters) restores mitochondrial turnover and reverses cognitive deficits in AD models [139]. Post-mortem human AD brains and AD-derived cellular models show that amyloid-β and phosphorylated tau interact with Drp1, PINK1 and parkin, with excessive mitochondrial fission and simultaneously disturbing autophagosome–lysosome fusion and lysosomal acidity. This results in accumulation of mitochondria-containing autophagic vacuoles and increased p62 in affected neurons [140].

Human pathological studies used phospho-ubiquitin (p-S65-Ub)—a PINK1-dependent “mitophagy tag” to distinguish between normal aging and neurodegeneration. In large cohorts of post-mortem human brains, p-S65-Ub-positive structures were detected and quantified in substantia nigra, hippocampus, amygdala, putamen and nucleus basalis. In neurologically normal individuals, p-S65-Ub density increased with age in the amygdala and nucleus basalis, consistent with an age-related rise or compensation in mitophagy signaling in these regions. In Lewy body disease, p-S65-Ub levels were significantly higher in the substantia nigra and hippocampus than in age-matched controls, correlating with Lewy body and neurofibrillary tangle burden. PRKN or PINK1 mutation carriers showed reduced or absent p-S65-Ub [141].

Basal mitophagy signaling increases with age in several human brain regions, but in Parkinsonian disorders, it becomes dysregulated, acquiring both region- and mutation-dependent failure of mitochondrial quality control.

Patient-derived organoid neuronal models have introduced an advanced modality to gain mechanistic insights into pathological aging. Human iPSC-derived dopaminergic neurons with PINK1 loss-of-function display normal mitochondrial morphology and preserved Parkin recruitment but show inhibited ionophore-induced mitophagy and reduced mitochondrial membrane potential, indicating a specific defect in the execution of mitophagy rather than in biogenesis or gross morphology [142]. Age-related neurodegenerative diseases such as AD and PD superimpose additional blocks at multiple levels of the mitophagy pathway (PINK1/Parkin signaling, receptor recruitment, autophagosome–lysosome fusion), disrupting mitochondrial network adaptability. If such disturbances are a cause or consequence of the disease, they need to be further explored.

In aging mice, senescent astrocytes accumulate morphologically damaged, fragmented mitochondria due to impaired mitophagy (PINK1–Parkin axis downregulation and increased fission), with reduced respiratory capacity and increased susceptibility to further mitochondrial stress [143]. Such changes can compromise metabolic and redox support to adjacent neurons and induce age-associated neuroinflammation. In human brain samples, 3D Electron microscopy (EM) and morphometry show mitochondrial malfunction and atrophy specifically in astrocytes—shorter, swollen mitochondria, reduced mitochondrial content, and astrocytic process atrophy—while neuronal mitochondria are comparatively preserved. This can be interpreted as an age-related shift where astrocyte mitochondrial failure undermines synaptic and metabolic support to neurons in the aged human cortex [144]. In human induced pluripotent stem cells (IPSC) models, astrocytes carrying POLG gene mutations (a gene that encodes the catalytic subunit of polymerase γ associated with mitochondrial inherited diseases) display loss of mitochondrial membrane potential I/IV deficiency, ATP failure and mtDNA depletion. Such astrocytes have an A1-like, reactive signature with neurotoxic effects on co-cultured neurons [145]. Limiting microglial mitochondrial fragmentation with the Drp1–Fis1 inhibitory peptide P110 stops A1 astrocyte conversion and protects neurons [146,147].

Metabolic coupling between oligodendrocytes and axons depends on intact oligodendrocyte mitochondria and their glycolytic/oxidative capacity. NMDA receptor activation in oligodendrocytes mobilizes GLUT1 and enhances glucose uptake and lactate delivery to axons, supporting axonal energy metabolism.

In demyelinating/neurodegenerative diseases, mtDNA double-strand breaks induced selectively in oligodendrocytes in mice lead to oligodendrocyte mitochondrial dysfunction, followed by axonal degeneration, even in the absence of oligodendrocyte loss. In patients with multiple sclerosis (MS), oligodendrocyte mitochondrial failure contributes to “dying-back” axonopathy and chronic neurodegeneration [148].

**Figure 5:**
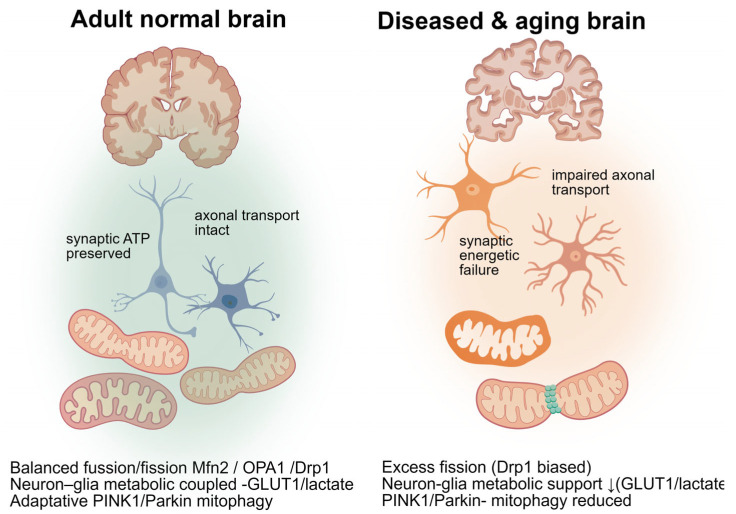
Brain mitochondrial network remodeling in aging. (**Left**): In the adult healthy nervous system, mitochondrial networks support intact axonal transport and synaptic function, with balanced fusion–fission regulation (MFN2/OPA1/DRP1), preserved ATP supply and Ca^2+^ buffering at synapses, and effective neuron–glia metabolic coupling (GLUT1-dependent glucose handling and lactate support). Adaptive PINK1/Parkin-mediated mitophagy contributes to mitochondrial turnover and network maintenance. (**Right**): During aging and in neurodegenerative/demyelinating contexts, mitochondrial network disruption is characterized by impaired axonal transport, fragmentation (DRP1-biased), reduced PINK1/Parkin mitophagy and diminished glial metabolic support, promoting synaptic energy failure and vulnerability to neurodegeneration. (Figure created with BioGDP.com).

While this evidence is disease-focused (MS and white-matter degeneration), it exemplifies how disrupted mitochondrial networks in myelinating glia can compromise glia–axon metabolic crosstalk, promoting neurodegeneration (Figure 5).

Mitochondrial connectivity operates in a tissue-dependent manner (Figure S1), contributing to adaptive responses that can propagate at the organismal level.

## 4. Targeting Mitochondrial Network to Prevent Age-Related Decline

Interventions such as physical exercise, caloric restriction or mimetics, mitophagy activation, and NAD^+^ boosting influence mitochondrial network organization, suggesting that reversibility of fragmented and dysfunctional states could be achieved. Mitochondrial network-targeted interventions can also be perceived through the framework of hormesis, in this context referred to as mitohormesis. From this perspective, mild and transient mitochondrial stress does not simply represent damage but can trigger adaptive programs that improve long-term cellular resilience. Caloric restriction, exercise, and some natural compounds appear to act, at least in part, by inducing a controlled redox and bioenergetic challenge that activates stress-response pathways rather than by directly suppressing ROS. Such responses may include activation of Nrf2-dependent antioxidant defense, AMPK/SIRT1/PGC-1α signaling, mitochondrial unfolded protein response programs, mitophagy, and compensatory mitochondrial biogenesis. At the network level, these adaptive responses may preserve fusion–fission balance, improve removal of dysfunctional mitochondrial nodes, limit chronic oxidative injury, and restore functional connectivity. It needs to be understood, however, that these effects are both dose- and context-dependent; the same compound may act as a beneficial hormetic stimulus at low or moderate exposure, but may become ineffective or even toxic at higher doses, especially in aged tissues with reduced metabolic reserve. Context and dose dependence appear to be essential for mitohormesis and should be taken into account from the perspective of mitochondrial protection [149,150].

### 4.1. Cardiac and Skeletal Muscle

Modulation of the SIRT1–AMPK–DRP1 pathway by natural or synthetic activators may rebalance mitochondrial fusion and fission, improve network connectivity, and support cardiac function in the aging heart [72]. mTOR inhibitors such as rapamycin activate ULK1 and upregulate autophagy, thereby increasing degradation of cytosolic cargo, including mitochondria [78]. In cardiac muscle, rapamycin has repeatedly been shown to induce autophagy and confer functional benefit, attenuation of pressure-overload hypertrophy, and improvement of diastolic function in aging animal models [79,80,81,82].

Skeletal muscle from well-trained older adults shows higher mitochondrial content and substantially more CRU–mitochondrial pairs than age-matched sedentary individuals [95]. Experimental studies in mice confirm this protective effect, showing that long-term endurance exercise largely prevents age-related CRU loss and CRU–mitochondrial uncoupling, thereby preserving SR Ca^2+^ release, mitochondrial Ca^2+^ handling, and redox homeostasis [96]. Aging skeletal muscle displays a hyper-fragmented mitochondrial network, whereas lifelong treadmill exercise normalizes network structure in wild-type, but not PGC-1α knockout, mice, supporting a role for PGC-1α in preservation of mitochondrial architecture [99]. In humans, lifelong high-volume endurance training is associated with greater mitochondrial volume and network connectivity, higher complex I+II respiration per muscle mass, better ANT1/2 and VDAC content, and preserved ADP sensitivity compared with untrained peers [99]. In observational studies, older adults with higher habitual physical activity also show better mitochondrial capacity, muscle quality, exercise efficiency, and physical performance [100,101].

In aged rodents, long-term endurance exercise restores mitochondrial protein levels and respiratory capacity, supporting a role for physical activity in mitochondrial biogenesis across the lifespan [108]. In humans, acute exercise markedly increases PGC-1α transcription and downstream mitochondrial gene expression in the vastus lateralis [109,110,111], while both high-intensity interval and conventional endurance training increase mitochondrial content, citrate synthase activity, cytochrome c oxidase activity, and overall oxidative capacity [112,113,114,115]. Physical activity also promotes mitophagy. In animal models, acute and chronic exercise increase AMPK–ULK1-dependent targeting of mitochondria to lysosomes; mitophagy flux declines when PGC-1α or Parkin is disrupted, whereas endurance training amplifies BNIP3-mediated signaling [120]. In humans, regular endurance training and an active lifestyle are associated with higher mitochondrial Parkin content and upregulated autophagy/mitophagy markers in skeletal muscle, while single bouts of endurance or resistance exercise acutely increase mitophagy signaling and mitochondria–lysosome interactions independently of age [121].

In aging muscle, caloric restriction mimetics and pharmacologic activation of fusion-supporting pathways, including PGC-1α agonists, NAD^+^ boosters, or urolithin A, may partially restore mitochondrial network integrity. Physical activity, especially regular endurance training, acts on several determinants of mitochondrial connectivity, including dynamic coupling, biogenesis, maintenance, and quality control.

Restoring mitochondrial network integrity through caloric restriction mimetics, or pharmacologic activation of fusion pathways (e.g., PGC-1α agonists, NAD^+^ boosters, or urolithin A) has shown promise in reversing the process.

### 4.2. Nervous System

In mice, microglial mitochondrial network disruption with excess fission impairs microglia–astrocyte crosstalk and, secondarily, astrocyte–neuron interactions, amplifying inflammatory neurodegeneration. In microglia, inhibition of excessive mitochondrial fragmentation through the Drp1–Fis1 inhibitory peptide P110 reduces the release of damaged mitochondria, prevents A1 astrocyte conversion, and protects neurons [147]. Intact oligodendrocyte mitochondria are essential for effective metabolic coupling with axons, as NMDA receptor activation promotes GLUT1 mobilization, glucose uptake, and lactate transfer to support axonal energy demands.

Regular endurance exercise has been shown to induce mitochondrial biogenesis programs in the brain, including increased expression of mitochondrial biogenesis regulators (PGC-1α/SIRT1-associated signatures) and mtDNA, in multiple brain regions.

In the aging brain, exercise also modulates mitochondrial network maintenance pathways: in old mice, training increased levels of the fission regulator DRP1 (without parallel changes in key fusion proteins in that study), thus remodeling mitochondrial dynamics as part of an adaptive response [151]. In APP/PS1 mice, a 12-week treadmill protocol improved mitochondrial function and cognitive performance while enhancing PINK1/Parkin-mediated mitophagy in the hippocampus [152].

In the nervous system, increasing attention has been given to natural compounds that may function as “mitohormetins”. Such compounds are able to induce a mild adaptive mitochondrial stress response, triggering endogenous defense pathways. Rather than acting only as direct ROS scavengers, these compounds may promote brain resilience by activating the Nrf2/ARE axis and its downstream targets such as HO-1, glutathione-related enzymes, and other stress-response mediators, while attenuating persistent NF-κB-driven inflammatory signaling. Mitohormetic compounds may support mitochondrial quality control, partially restore biogenesis and mitophagy, reduce accumulation of damaged mitochondria, and improve redox balance in neurons and glial cells. Polyphenols and related bioactive nutrients have been discussed in this context, particularly in aging and neurodegeneration, where chronic oxidative stress, low-grade inflammation, lysosomal dysfunction, and impaired mitophagy co-exist [153,154,155]. At the same time, current evidence remains mostly preclinical and should be interpreted cautiously, because beneficial effects depend on concentration, duration of exposure, bioavailability, blood–brain barrier penetration, and the pre-existing metabolic state of the tissue. Thus, in nervous system disorders, mitohormetins may function not as mere antioxidants, but as context-dependent modulators of endogenous mitochondrial response.

Physical exercise can act at multiple levels—biogenesis, dynamics remodeling, and mitophagy—to partially counteract age-associated mitochondrial network fragmentation and sustain neuroglial metabolic support. The emerging literature suggests that natural and nutrient-derived compounds may complement exercise and caloric restriction-like interventions by acting on overlapping adaptive pathways [156,157,158]. In aging and age-related disorders, these responses have been linked mainly to activation of Nrf2-centered antioxidant programs, modulation of SIRT1/AMPK-related signaling, attenuation of persistent NF-κB-driven inflammatory signaling, and support of mitochondrial quality control, including biogenesis and mitophagy (Table 1). Their relevance to mitochondrial network biology lies not only in reducing oxidative burden but in reshaping how cells respond to stress through coordinated effects on redox signaling, inflammatory tone, mitochondrial turnover, and energetic plasticity. In this sense, the therapeutic interest of mitohormetins is not the complete suppression of mitochondrial stress, but the induction of a controlled adaptive response able to preserve network coherence.

## 5. Mitochondrial Networking in Aging: A Systems-Theory Perspective

The framework proposed here integrates the molecular- and tissue-level observations discussed above. Intracellular fusion–fission dynamics, mitophagy–biogenesis coupling, and cristae architecture determine node quality and local connectivity, whereas intercellular mitochondrial transfer through tunneling nanotubes, extracellular vesicles, and fusion events extends this interaction to tissue level. Specific changes in heart, skeletal muscle, and nervous system can be viewed as distinct expressions of the same underlying network principles, governed by geometry, energetic demand, and adaptability.

Recent research increasingly supports the view that mitochondria are not isolated organelles, but components of a multiscale network operating within cells, between cells, and across tissues. From a systems perspective, this network can be regarded as a complex adaptive structure whose global behavior—maintenance, resilience, response to perturbation, or decline—emerges from local interactions and feedback loops.

Quantitative approaches borrowed from graph theory, percolation theory, active matter, and nonequilibrium statistical mechanics have already been used to describe mitochondrial branching, connectivity, fusion–fission kinetics, and dynamic remodeling under energetic and mechanical constraints. These studies show that mitochondrial networks can occupy different organizational regimes, ranging from fragmented to reticular or hyperconnected and can shift between them when fusion–fission balance, membrane properties, or metabolic state change [159,160,161,162,163,164,165].

In this context, mitochondrial networking should be understood not only as morphology or organelle trafficking but as a systems property that includes topology, dynamics, and information transfer. Network behavior depends not only on the number and quality of mitochondrial nodes but also on how effectively signals, metabolites, membrane potential changes, and damage responses propagate through the system.

Within this framework, mitochondrial network coherence may be proposed as a composite operational order parameter. It integrates several measurable features, including network topology, energetic coupling, quality-control dynamics, and intercellular exchange. Coherence may be approximated by parameters such as fusion–fission balance and branching indices, cristae junction integrity and respiratory super complex stability, spatial and temporal distribution of ΔΨm, the balance between mitophagy and biogenesis, and the density and directionality of intercellular mitochondrial transfer (Figure S2 Supplementary material).

Loss of mitochondrial coherence does not necessarily require physical disconnection. Networks may remain morphologically continuous while losing coordinated response and adaptive reconfiguration. Hyperfusion, cristae swelling, or arrested fusion–fission dynamics may therefore represent rigid, low-plasticity network states rather than preserved integrity. From this perspective, aging is not a single morphological endpoint but a gradual shift toward states of reduced coherence and diminished adaptability, regardless of the precise mitochondrial shape adopted.

This view does not replace classical damage-based theories of aging but reframes them. Damage to DNA, proteins, lipids, or mitochondrial membranes remains important, yet its consequences depend on where it occurs in the network and how efficiently the network compensates. In a well-connected and adaptive system, local defects may be buffered through fusion-mediated complementation, selective mitophagy, and redundancy in intercellular exchange. As connectivity declines—through reduced fusion, impaired mitophagy, altered transport, loss of CRU–mitochondrial coupling, or disturbed glia–neuron and muscle–organ communication—the same degree of local damage may have much broader functional consequences. In this sense, mitochondrial networking helps define the resilience landscape of the organism. Aging could therefore be interpreted as a progressive loss of mitochondrial network coherence. Youthful and aged states may thus correspond to different dynamic regimes of the mitochondrial network.

Endurance exercise, caloric restriction mimetics, mitophagy activators, and fusion-supporting interventions can restore aspects of mitochondrial architecture and function in aged tissues. In systems terms, such interventions improve node quality, enhance connectivity, and reinforce control loops such as AMPK–SIRT–PGC-1α signaling and mitophagy–biogenesis coupling. The requirement for stronger or more sustained intervention later in life suggests hysteresis: once the system settles into an aged low-coherence state, returning it toward a more connected regime becomes more difficult than maintaining it earlier in life. If restoration of coherence leads to functional improvement, then loss of coherence is not only a consequence of aging but a contributor to it.

At the intracellular level, this corresponds to a shift from a connected and self-correcting mitochondrial network toward one that is fragmented or rigid and poorly repaired. At the intercellular level, aging involves deterioration of communication routes such as TNTs, extracellular vesicle exchange, fusion events, and broader metabolic coupling between neighboring cells. At the tissue and organism level, cumulative loss of mitochondrial connectivity contributes to poorer energy distribution, slower adaptation to stress, and weaker integration of systemic responses.

This loss of coherence may contribute to classical aging phenotypes, including frailty, sarcopenia, immunosenescence, and neurocognitive decline, while also remaining at least partly modifiable. The hypothesis could be tested in several ways: by identifying thresholds of connectivity loss through longitudinal analysis of network topology and synchrony; by defining marker panels that reflect node quality, mesoscale architecture, and intercellular communication; by visualizing cross-tissue mitochondrial coupling using tissue-specific reporters, in vivo imaging, and circulating biomarkers; and by comparing interventions according to their ability to restore multiscale connectivity rather than isolated molecular targets. Interventions restoring this integrated connectivity may have an increased impact on health span than those acting on single pathways alone.

Viewing mitochondria as elements of a multiscale adaptive network shifts attention from isolated organelle damage to the coherence of energy and information flow across cells, tissues, and the organism. In this view, aging reflects progressive failure of the mitochondrial network: partly secondary to damage, but also causally involved in the loss of systemic robustness. Its partial reversibility through lifestyle and pharmacological intervention suggests that biological age depends not only on time but also on the extent to which mitochondrial networks remain functionally connected and adaptative.

## 6. Conclusions and Perspectives

Mitochondria are dynamic networked organelles whose organization influences cellular and tissue homeostasis. Fusion–fission balance, mitophagy–biogenesis coupling, organelle positioning, and intercellular mitochondrial exchange account for functional connectivity in myocardia, skeletal muscle, and the nervous system.

A central concept emerging from this review is that aging may reflect progressive loss of mitochondrial network coherence. Structural fragmentation, impaired cristae organization, defective trafficking, reduced quality control, and disturbed metabolic communication all decrease the capacity of mitochondrial networks to support tissue adaptability. Conversely, interventions such as exercise, caloric restriction or its mimetics, mitophagy-enhancing strategies, and selected network-supporting pharmacologic or nutritional interventions may partially restore mitochondrial organization and function even in aged tissues. The growing interest in hormesis and mitohormesis offers an unprecedented modality for influencing adaptative responses. So far, evidence points to the fact that effects are highly dose-, time-, and context-dependent, and the boundary between beneficial remodeling and additional stress may shift with age and disease.

This review also has several limitations. First, the field remains heterogeneous, with evidence derived from diverse experimental systems, species, and tissues that are not always directly comparable. Second, many currently available data describe mitochondrial morphology or single pathways, while direct measurements of functional network coherence remain limited. Third, evidence for intercellular mitochondrial exchange and its contribution to physiological versus pathological aging is still uneven for different body tissues and is much stronger in experimental models than in humans.

Future work should therefore move beyond isolated molecular markers toward integrated assessment of mitochondrial networks across hierarchical systems. Priorities include development of standardized metrics for mitochondrial connectivity and quality control, longitudinal imaging approaches, spatial omics strategies, circulating biomarkers reflecting mitochondrial turnover and intercellular exchange, and computational models able to relate local organelle behavior to tissue-level function. In parallel, interventions including exercise, caloric restriction mimetics, mitophagy activators, and selected mitohormetic compounds should be compared not only for pathway activation, but also for their ability to restore multiscale mitochondrial coherence. Such an approach may help clarify whether mitochondrial network remodeling is merely an effect of aging or a tractable determinant of tissue resilience and health span.

Future work integrating imaging, spatial omics, and computational modeling may help identify markers of network integrity and guide strategies to preserve resilience during aging.

## Figures and Tables

**Table 1:** Representative natural compounds with putative mitohormetic activity and their effects on mitochondrial activity.

Compound/Class	Pathways Mainly Upregulated	Pathways Mainly Downregulated/Restrained	Reported Mitochondrial Network-Related Effects	Ref
Isothiocyanates (e.g., sulforaphane)	Nrf2/ARE, HO-1, glutathione-related enzymes, phase II detoxification programs	Persistent NF-κB-associated inflammatory signaling	Antioxidants may reduce ROS burden, favors mitochondrial quality control and preservation of functional integrity	[149,150]
Polyphenols (e.g., resveratrol, curcumin, quercetin, EGCG)	Nrf2, SIRT1, AMPK, PGC-1α-associated programs	NF-κB, chronic inflammatory cytokine signaling	Support mitochondrial biogenesis, redox resilience, and metabolic adaptation; effects are dose-dependent	[149,153]
Hormetic phytochemicals with transient mitochondrial inhibition (example: chrysin)	Endogenous stress-defense pathways, antioxidant adaptation, survival signaling	Sustained oxidative injury after adaptive response is established	Experimental support for the concept that mild mitochondrial perturbation can induce stress resistance and longevity-associated adaptation	[155,156,157]
Functional food nutrients/neuronutrients	Nrf2, cytoprotective and redox resilience genes, neuroprotective signaling	NF-κB-linked neuroinflammatory tone	Maintains redox balance, mitophagy competence, and resistance to mitochondrial dysfunction in neural cells	[153,155,156]
Natural compounds with anti-aging hormetic profile (broad class)	Nrf2-centered adaptive defense, stress-response enzymes, resilience	Oxidative and inflammatory overactivation when adaptation is achieved	Preservation of mitochondrial homeostasis, redox balance, and quality control, translation context dependent	[149,157]

## Data Availability

No new data were created or analyzed in this study. Data sharing is not applicable to this article.

## Supplementary Materials

The following supporting information can be downloaded at: https://www.mdpi.com/article/10.3390/ijms27083557/s1.

## Abbreviations

The following abbreviations are used in this manuscript:

AMPK	AMP-activated protein kinase
ATP	adenosine triphosphate
βAPP	beta-amyloid precursor protein
BNIP3	BCL2 interacting protein 3
DAMPs	damage-associated molecular patterns
DRP1	dynamin-related protein 1
GTP	guanosine triphosphate
EM	electron microscopy
FoxOs	Forkhead box O family of transcription factors
GLUT1	glucose transporter type 1
IκB-α	inhibitor of kappa B-alpha
iPLA2β	calcium-independent phospholipase A2 beta
LC3-I	microtubule-associated protein 1A/1B-light chain 3-I
LC3-II	microtubule-associated protein 1A/1B-light chain 3-II
MFN1/2	mitofusin 1 and 2
NMDA	N-methyl-D-aspartate
NF-κB	nuclear factor-kappa B
NRF1/2	nuclear respiratory factor 1 and 2
OGG1	8-oxoguanine DNA glycosylase
OPA1	optic atrophy 1
p62	sequestosome 1 (SQSTM1)
PCr	phosphocreatine
PGC-1α	peroxisome proliferator-activated receptor gamma coactivator 1-alpha
RalA–M-Sec–exocyst complex	molecular machinery regulating membrane trafficking and remodeling, including tunneling nanotube formation and exocytosis
ULK1–ATG13–FIP200	mammalian autophagy-initiating kinase complex composed of ULK1, ATG13, and FIP200
TFAM	mitochondrial transcription factor A
TNF-α	tumor necrosis factor alpha
Ser616	serine 616 residue in dynamin-related protein 1
SOD2	superoxide dismutase 2
