# Supplementary material for: Mitochondrial Network Dynamics in Aging: Cellular Mechanisms, Intercellular Communication, and Their Impact on Tissue Adaptability
Source: Int J Mol Sci. 2026 Apr 16;27(8):3557. doi: 10.3390/ijms27083557 (PMC13115720; doi:10.3390/ijms27083557)
Supplement: Supplementary file 1 [file ijms-27-03557-s001.zip › ijms-4184041-supplementary.pdf]

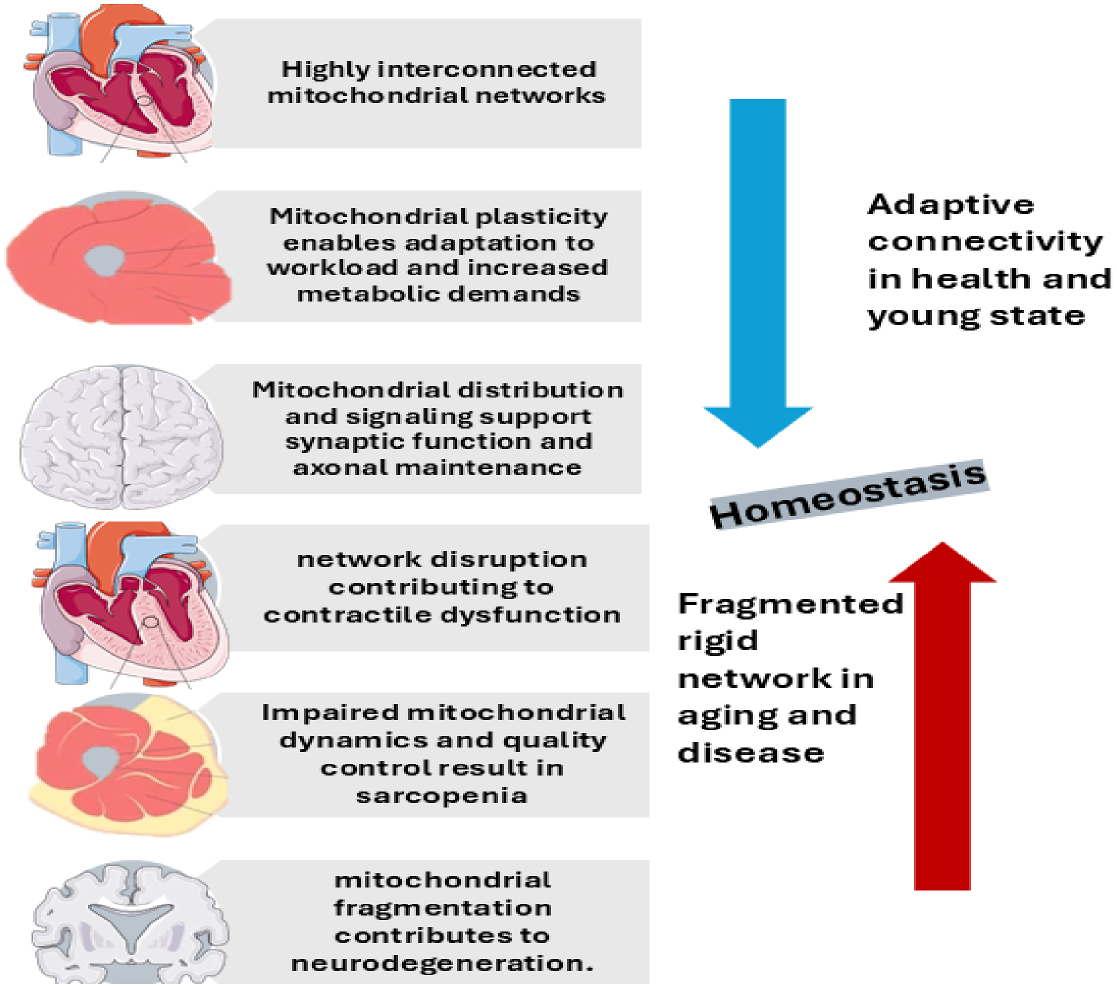

Figure S1. Tissue-specific manifestations of mitochondrial network function. Simplified schematics illustrate how shared mitochondrial principles are implemented in a tissue-dependent manner. Cardiac tissue: highly interconnected mitochondrial networks support continuous ATP demand and excitation–contraction coupling, with disruption contributing to contractile dysfunction and aging-related decline. Skeletal muscle: mitochondrial plasticity enables adaptation to workload and metabolic stress; impaired dynamics and quality control are associated with sarcopenia and reduced regenerative capacity. Nervous tissue: mitochondrial distribution and signaling support synaptic function and axonal maintenance, while age-associated fragmentation contributes to neurodegeneration.

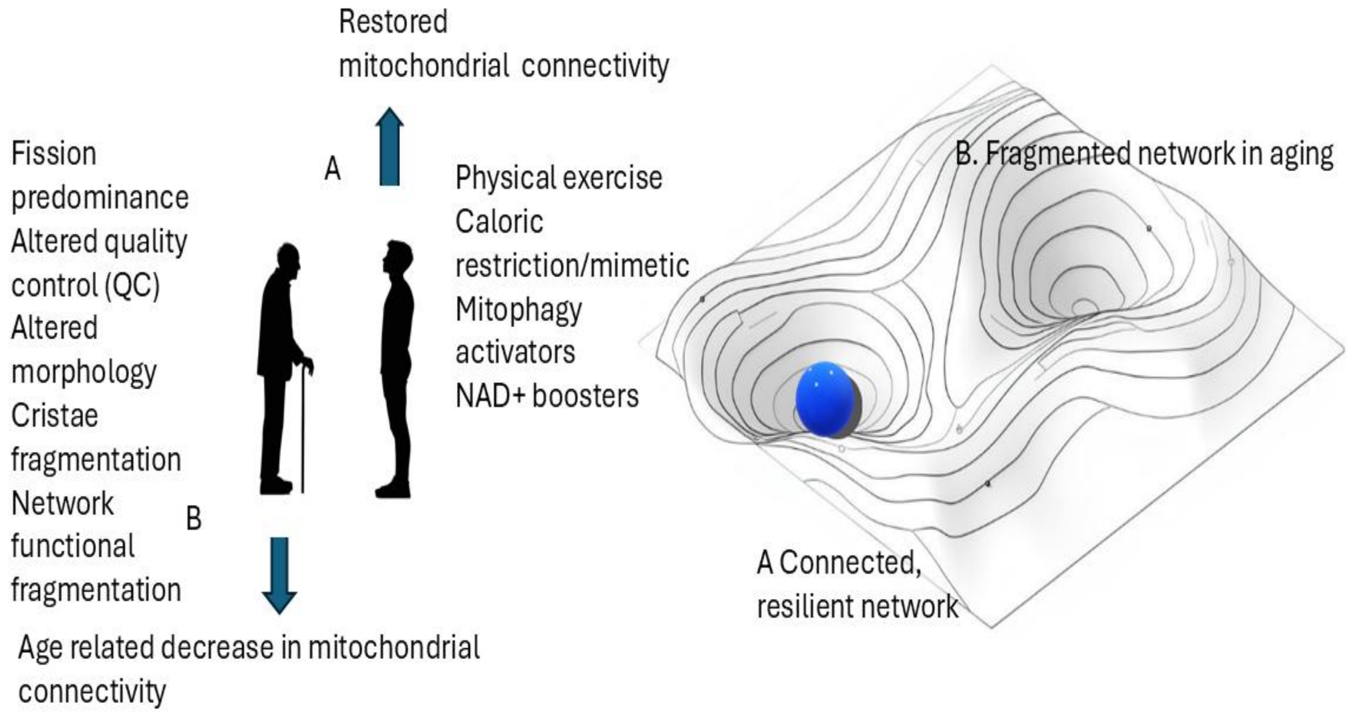

Figure S2. Molecular and dynamic mechanisms governing mitochondrial network behavior and loss of mitochondrial coherence as aging trajectory. Aging is depicted as a progressive shift between attractor states within a dynamic landscape of mitochondrial network organization. Key pathways regulating network integrity are highlighted, including fusion–fission dynamics, mitophagy–biogenesis coupling, and stress-responsive signaling. When these processes are balanced, network coherence supports bioenergetic efficiency, redox signaling, and controlled inflammatory tone; disruption promotes fragmentation and functional decline. Partial reversibility is proposed through interventions that target drivers of network disorganization.
